# The overlap between randomised evaluations of recruitment and retention interventions: An updated review of recruitment (Online Resource for Recruitment in Clinical triAls) and retention (Online Resource for Retention in Clinical triAls) literature

**DOI:** 10.1177/17407745241238444

**Published:** 2024-04-04

**Authors:** Anna Kearney, Laura Butlin, Taylor Coffey, Thomas Conway, Sarah Cotterill, Alison Evans, Jackie Fox, Andrew Hunter, Sarah Inglis, Louise Murphy, Nurulamin M Noor, Terrie Walker-Smith, Carrol Gamble

**Affiliations:** 1 Department of Health Data Science, University of Liverpool, Liverpool, UK; 2 Centre for Trials Research, Cardiff University, Cardiff, UK; 3 Health Services Research Unit, University of Aberdeen, Aberdeen, UK; 4 HRB-TMRN, Trial Methodology and Evidence Synthesis, University of Galway, Galway, Ireland; 5 Centre for Biostatistics, Division of Population Health, Health Services Research & Primary Care, The University of Manchester, Manchester, UK; 6 Cardiff University, Cardiff, UK; 7 MRC Clinical Trials Unit, University College London, London, UK; 8 School of Nursing & Midwifery, University of Galway, Galway, Ireland; 9 Tayside Clinical Trials Unit (TCTU), University of Dundee, Dundee, UK; 10 Health Research Institute, Department of Nursing Studies & Midwifery, University of Limerick, Limerick, Ireland; 11 Bristol Trials Centre, University of Bristol, Bristol, UK; 12 Liverpool Clinical Trials Centre, University of Liverpool, Liverpool, UK

**Keywords:** Recruitment, retention, methodological research, SWAT

## Abstract

**Background:**

The Online Resource for Recruitment in Clinical triAls (ORRCA) and the Online Resource for Retention in Clinical triAls (ORRCA2) were established to organise and map the literature addressing participant recruitment and retention within clinical research. The two databases are updated on an ongoing basis using separate but parallel systematic reviews. However, recruitment and retention of research participants is widely acknowledged to be interconnected. While interventions aimed at addressing recruitment challenges can impact retention and vice versa, it is not clear how well they are simultaneously considered within methodological research. This study aims to report the recent update of ORRCA and ORRCA2 with a special emphasis on assessing crossover of the databases and how frequently randomised studies of methodological interventions measure the impact on both recruitment and retention outcomes.

**Methods:**

Two parallel systematic reviews were conducted in line with previously reported methods updating ORRCA (recruitment) and ORRCA2 (retention) with publications from 2018 and 2019. Articles were categorised according to their evidence type (randomised evaluation, non-randomised evaluation, application and observation) and against the recruitment and retention domain frameworks. Articles categorised as randomised evaluations were compared to identify studies appearing in both databases. For randomised studies that were only in one database, domain categories were used to assess whether the methodological intervention was likely to impact on the alternate construct. For example, whether a recruitment intervention might also impact retention.

**Results:**

In total, 806 of 17,767 articles screened for the recruitment database and 175 of 18,656 articles screened for the retention database were added as result of the update. Of these, 89 articles were classified as ‘randomised evaluation’, of which 6 were systematic reviews and 83 were randomised evaluations of methodological interventions. Ten of the randomised studies assessed recruitment and retention and were included in both databases. Of the randomised studies only in the recruitment database, 48/55 (87%) assessed the content or format of participant information which could have an impact on retention. Of the randomised studies only in the retention database, 6/18 (33%) assessed monetary incentives, 4/18 (22%) assessed data collection location and methods and 3/18 (17%) assessed non-monetary incentives, all of which could have an impact on recruitment.

**Conclusion:**

Only a small proportion of randomised studies of methodological interventions assessed the impact on both recruitment and retention despite having a potential impact on both outcomes. Where possible, an integrated approach analysing both constructs should be the new standard for these types of evaluations to ensure that improvements to recruitment are not at the expense of retention and vice versa.

## Introduction

Recruiting and retaining participants have been identified as two of the most important challenges to clinical trials and are key priorities for methodological research.^1^ Failure to recruit and retain sufficient numbers of participants can lead to lack of statistical power to analyse treatment effects. Failure to retain participants can also introduce bias if the reason for loss is unbalanced across trial arms or is connected to the treatments under investigation.^2^

Challenges with recruitment have been well reported^3–5^ and while some progress has been made, it continues to affect a substantial number of trials. Only 61% of National Institute for Health and Care Research (NIHR) trials funded between 2017 and 2020 achieved their original target with 37% extending the recruitment period to meet either the original or a revised target.^6^ Of the two challenges, recruiting sufficient numbers of participants has often been seen as a primary pressing concern^7^ as it is regularly tracked by funders, with recruiters and healthcare sites often incentivised to achieve pre-planned targets.^8,9^ However, there is growing recognition that retention deserves equal focus given that efforts to achieve recruitment targets are wasted if participants are not subsequently retained in the study.^7,10^ Analysis of retention rates suggests that between 10% and 20% of participants are not included in the primary analysis^6^ due to participants no longer being involved in the study or their primary outcome not being available. However, some research areas, study designs and participant characteristics have been associated with higher levels of attrition.^11–14^

While patient and public involvement within trial design can help mitigate some of these challenges, evidence for effective interventions is needed to improve both recruitment and retention rates. Published literature reporting challenges and potential solutions have been growing over the last decade, although much of it is anecdotal. Initiatives have encouraged the use of methodological research studies nested within a trial (SWATs) to provide evidence for effective strategies.^15,16^ Yet, methodological research is not well indexed so identifying relevant studies through traditional databases is challenging and time-consuming. Consequently, the Online Resource for Recruitment in Clinical triAls (ORRCA) was launched in 2016^17^ to regularly map and organise literature on recruitment strategies into a free, searchable, online database (www.orrca.org.uk). This was expanded in 2019 to include a second database, the Online Resource for Retention in Clinical triAls (ORRCA2), covering retention literature.^18^

Although contained within the same website, the initial literature reviews and subsequent updates for the ORRCA and ORRCA2 databases were conducted separately but in parallel. However, recruitment and retention of participants are interconnected and need to be considered together during trial design and conduct. Recruitment targets can be inflated to allow for anticipated attrition rates to ensure enough participants with analysable data are available to reach a statistical conclusion. If follow-up schedules are considered to be too burdensome by patients, this may negatively impact both recruitment and retention rates.^19^ Due to their interconnected nature, it is important that researchers consider potential positive and negative consequences of any recruitment or retention strategies on the reciprocal challenge.

Work has explored the scope and coverage of randomised evaluations for either recruitment or retention interventions.^20–22^ However, dual assessment of recruitment and retention has been overlooked until more recently. A systematic review has identified 10 randomised studies of recruitment interventions that also assessed retention outcomes.^23^ Authors recommended recruitment studies should consider dual assessment to improve the evidence base, but it is unclear to what extent dual assessment is appropriate or feasible. This publication aims to report and compare recent updates of ORRCA and ORRCA2 to identify any changes in the literature and to explore the potential for dual assessment within the context of recruitment and retention interventions being evaluated.

## Methods

Updates for the recruitment (ORRCA) and retention (ORRCA2) database were conducted separately but used similar methodology which has been previously described.^17,18^ A brief outline is provided here with descriptions of any changes made from the original methodology.

### Search strategies and identification of the literature

The original search strategy used in the development of the ORRCA database^17^ was adjusted in a subsequent database update that has not been published. The revisions created a single search strategy that was then adapted for use across the databases described below. The ORRCA update reported here used the revised strategy (Supplementary File). No changes were made to the ORRCA2 search strategy previously reported.^18^ Searches of Medline (Ovid), CINAHL (EBSCO), PsycINFO (EBSCO), Scopus, Web of Science Core Collection (SCI-expanded, SSCI, CPCI-S, CPCI-SSH, ESCI) and the Cochrane library (CENTRAL) were run between 9 and 10 April 2020 (ORRCA) and in January 2020 (ORRCA2). Both searches were restricted to papers published between 2018 and 2019 as ORRCA and ORRCA2 already contained papers published up to the end of 2017. Hand searches of key systematic reviews identified within each update were undertaken (Supplementary File).

The full text of eligible articles that were categorised as ‘randomised evaluations’ were checked to see whether any secondary outcomes meant they should also be included in the other database. For example, a nested study evaluating the effect of patient information leaflets on recruitment rates that also assessed the effect on retention rates.

The study inclusion and exclusion criteria remain unchanged. Studies describing or evaluating activities, study designs or interventions aimed at addressing recruitment/ retention within health research studies were included. In line with previously reported ORRCA/ORRCA2 methods, the search strategies were designed to focus on recruitment/retention within host studies classed as randomised controlled trials. However, articles reporting recruitment/retention within other health research designs such as cohort studies, longitudinal surveys and non-randomised pilot studies that were returned by the searches were included as they are likely to contain sources of transferable knowledge and ideas for randomised controlled trials.

Eligible recruitment articles explored activities and methods that impacted the recruitment process. For example, methods for recruitment rate predication, engagement of research sites, identification of potential participants, information provision and the consent process. Articles exploring reasons for study participation were also included.^17^

Eligible retention articles explored activities and methods that impacted on the willingness or ability of consented participants to complete planned data collection. This also included exploration of reasons for early study withdrawal or ongoing participation. Articles only exploring adherence to a clinical intervention were not eligible.^18^

### Screening and data extraction

Records returned by the searches for each update were imported into Endnote and duplicates were removed. For the recruitment search results, an automated screening process was used to rank records returned by searches in order of relevance with the top 45% of records screened manually as per the algorithm.^24^ ORRCA2 had a full manual screening process to organise the retention search results.

As previously described, the title and abstract of returned records for both ORRCA and ORRCA2 were single screened by A.K. and a team of volunteers. Volunteers were provided with training and written guidance and 10% of abstracts were independently checked by a second reviewer (A.K.) as part of quality assurance measures. Full texts were obtained for all potentially relevant articles and were assigned a primary reviewer. Where the primary reviewer was not A.K. (50%), A.K. acted as a secondary reviewer to ensure data extraction consistency. Queries or disagreements were resolved through discussion with a third reviewer (C.G.).

Eligible articles were categorised against all relevant domains within the recruitment or retention frameworks^17,18^ (Supplementary File) and into one of the following types of evidence: Randomised evaluations of recruitment/retention strategies; Non-randomised evaluations of recruitment /retention strategies (e.g. pre-/post-test); Application of recruitment/retention strategies without comparative evaluation; Observations of factors affecting recruitment/retention without presenting a formal strategy (e.g. studies exploring patient-reported reasons for early study withdrawal).

Domains within the recruitment framework (Supplementary File) were subjectively coded by one author (A.K.) as having a potentially high, low or unclear impact on retention and vice versa based on their personal experience and knowledge of the recruitment and retention literature.

### Analysis

Descriptive statistics were used to summarise the recruitment and retention literature. The frequencies of recruitment and retention domains were calculated and presented as a percentage of all articles in the respective databases and in a subset of articles categorised as randomised evaluations. Domain frequencies of articles categorised as randomised evaluations were also assessed according to subgroups: studies only included in the recruitment database; studies only included in the retention database; and studies included in both databases. Analysis was conducted in SAS 9.4.

## Results

### Eligible records

**Figure 1.**
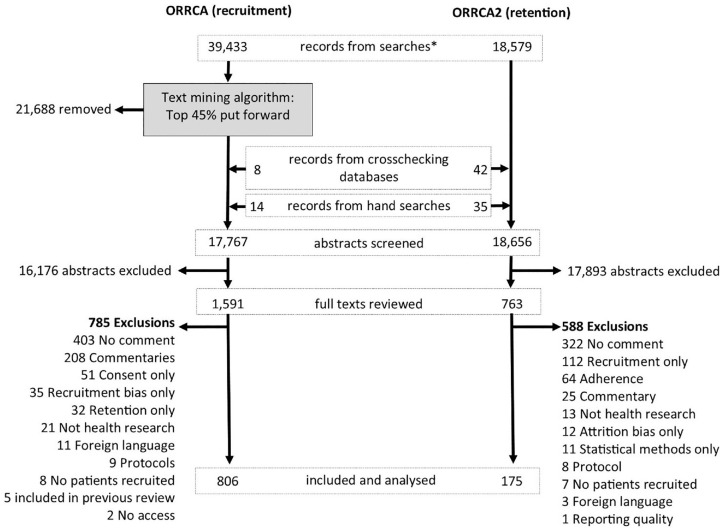
Identification of eligible studies from 2018 to 2019. *Unique records following automatic removal of duplicates within endnote.

Searches of the recruitment literature identified 39,433 unique records which were ranked by the text mining algorithm according to relevance (Figure 1). In total, 17,767 abstracts were manually screened for the recruitment update of which 806 were eligible. For the retention literature update, 18,656 records were screened of which 175 articles were eligible for inclusion. A total of 4813 articles were available on the recruitment database and 1338 on the retention database as of 20 February 2023.

### Cohort characteristics

Most newly identified studies included within ORRCA or ORRCA2 were conducted within America and Europe (Supplementary Table 1). In addition, the most common host design was randomised controlled trials. Studies that evaluated recruitment were more likely to recruit participants from secondary or tertiary care while studies evaluating retention were more likely to recruit from other sources (e.g. community events, social media etc). Both the recruitment and retention studies were most frequently conducted within cancer (212/806, 26% and 21/175, 12%, respectively).

### Recruitment and retention research design

The retention literature was twice as likely to include randomised evaluations (32/175, 18%) than the recruitment literature (70/806, 9%) (Table 1). The most frequent outcome measure for both databases was the numbers of participants recruited and retained. However, the percentage of studies assessing this was significantly higher for the retention database (139/175, 79% compared to 368/806, 46%). Recruitment literature was more likely to focus on evaluating patient perspectives (e.g. willingness to participate and reasons for participation decisions), whereas the retention literature was more likely to assess representativeness and questionnaire response rate. Recruitment literature was also more likely to assess cost of the intervention, but this was still low for both recruitment and retention (41/806, 5% and 2/175, 1%, respectively)

**Table 1.** Methods and outcomes for the nested research on participant recruitment or retention.

Research evidence type	Recruitment (n = 806)	Retention (n = 175)
Randomised evaluation	70 (9%)	32 (18%)
Non-randomised evaluation	93 (12%)	26 (15%)
Application	235 (29%)	48 (27%)
Observation	408 (51%)	69 (39%)
Research Methods	Recruitment (n = 806)	Retention (n = 175)
Case report	228 (28%)	67 (38%)
Survey	189 (23%)	11 (6%)
Qualitative interviews	155 (19%)	12 (7%)
Secondary analysis of a case report	97 (12%)	34 (19%)
Focus Groups	63 (8%)	7 (4%)
Systematic review and reviews	49 (6%)	18 (10%)
Nested RCT	42 (5%)	22 (13%)
Pre/Post Test	35 (4%)	5 (3%)
Other	29 (4%)	4 (2%)
Randomised study	26 (3%)	6 (3%)
Vignettes	25 (3%)	0 (0%)
Nested case control	13 (2%)	2 (1%)
Workshop proceedings	5 (1%)	0 (0%)
Research outcomes	Recruitment (n = 806)	Retention (n = 175)
Numbers recruited/retained	368 (46%)	139 (79%)
Reasons for participation or refusal/Reasons for withdrawal	214 (27%)	10 (6%)
Other	145 (18%)	17 (10%)
Willingness to Participate	132 (16%)	n/a
Representativeness	58 (7%)	26 (15%)
Recruitment/Retention Cost	41 (5%)	2 (1%)
No Evaluation	28 (3%)	12 (7%)
Recruitment Rate	16 (2%)	n/a
Questionnaire response rate	10 (1%)	21 (12%)

### Recruitment domains

Eligible papers were categorised according to the domains specified within the respective frameworks (Supplementary File). Recruitment literature reported a median of 2 domains per paper (interquartile range (IQR) [2,3] min 1, max 16). Most frequently reported domains were *B1: Trial acceptability*, *B10/C3: Barriers and facilitators (pre-trial/trial conduct), C7: Identification of participants* and *D7: Trial marketing* (Supplementary Figure 1). No newly identified studies explored *B6: Sample size estimation* and *B9: importance of outcomes to recruiters*. Of the studies categorised as randomised evaluations, assessment focussed predominantly on domains exploring the recruitment information and consent process: *D2: Participant information sheets* (24/70, 34%); *D3: Delivery of recruitment information* (19/70, 27%); *D4: Alternative technology (e.g. web, multimedia)* (14,70, 20%); *C8: Consent process* (11/70, 16%); *F1: Participant monetary incentives* (11/70, 16%).

### Retention domains

Papers from the retention literature reported a median of three domains per paper (IQR [1,4] min 1, max 17). Most frequently assessed strategies were aimed at participants (domains B1-B12), along with *A3: Data collection location* (Supplementary Figure 2). In this cohort, 20/175 (11%) assessed the impact of recruitment on retention (E3). No studies assessed the effects of *C3: Non-monetary incentives (Sites), D1: Monitoring approaches* or *E7: Run in period*. Of the studies categorised as randomised evaluations, the most frequently assessed domains were *B1: Reminders (participants)* (10/32, 31%), *B2: Monetary incentives (participants)* (10/32, 31%), *A3: Data collection methods* (6/32, 19%) and *E3: impact of recruitment* (6/32, 19%).

### Crossover of databases

Two new papers that had not been identified by the searches were added to the retention database following cross-checking of papers categorised as randomised evaluations. No new papers were identified for the recruitment database. Overall, 35 articles from the update were included in both databases of which 13 were categorised as randomised evaluations. Three of the 13 were systematic reviews evaluating a range of strategies (Supplementary Table 2) and 10 were randomised studies evaluating a methodological intervention. Of those 10 studies (Table 2), four evaluated recruitment information material, three monetary incentives with the others evaluating opt in/opt out recruitment, passwords, format of data collection and site initiation training.

**Table 2.** Randomised recruitment/retention studies that appear in both databases.

ID	Intervention	Retention assessment	Domains	
			Recruitment	Retention
153	Five different monetary incentive strategies (Pilot)	Data completion at weeks 2, 4, 6	F1	B2
218	Five different monetary incentive strategies (Main study)	Data completion at weeks 2, 4, 6	F1	B2
232	Opt in vs opt out recruitment strategy	Attendance at 3-month and 6-month follow-up visit	A3	E3
1585^a^	Handwritten vs printed name on invitation letter	Numbers retained at 3 months (i.e. returned monthly data)	D3	E3
2387	Inappropriate vs appropriate Password and high vs low reading age password	6-month follow-up rate	C3	B5; B6
2538^a^	Standard printed information leaflet alone or with multimedia resource	6- and 12-month retention rates	D2; D4	E3
3801	Paper vs online questionnaires	3-month follow-up questionnaire rates	B11	A1; A3
4231	Usual consent process alone or with standardised video on importance of follow-up	Completed the extended follow-up period: 3 years after renewed consent (five years after initial consent)	D4; D2	E3
4531	Face to face or online set up meetings for research sites	Questionnaires returned at week 6 and 52	D1	C9
5609	Posted invitation pack including a pen vs no pen	Retention at 3 months (returned 3 months of data)	G1-pen with recruitment	E3

a Added to the retention database following a cross-check of randomised evaluations across the two databases.

Of the studies categorised as randomised evaluation in the updates, 76/89 (85%) were only in one of the databases. Three of these were systematic reviews and 73 were randomised evaluations of methodological interventions. Of the randomised studies evaluating recruitment interventions that were not in the retention database, 48/55 (87%) reported domains associated with content and mode of consent information (D2, D3, D4) (Table 3). Similarly, randomised studies of retention interventions not in the recruitment literature most frequently reported domains of *B1: Participant reminders* (8/18, 44%), *B2: Participant monetary incentives* (6/18, 33%) and *A3: Data collection location and methods* (4/18, 22%) (Table 4).

**Table 3.** Frequency of recruitment domains reported in randomised studies of recruitment interventions that did not also assess retention.

Recruitment domain^a^	Domain category	Domain	Frequency N = 55	Likely impact on retention
**D2**	Recruitment Information	Participant Information Sheet and Consent Form	20 (36%)	High
**D3**	Recruitment Information	Delivery of information e.g. face to face, by post, etc.	17 (31%)	High
**D4**	Recruitment Information	Technology e.g. text, email, website etc	11 (20%)	High
**C8**	Trial Conduct	Consent Process	9 (16%)	High
**D6**	Recruitment Information	Non-trial specific information	6 (11%)	High
**F1**	Incentives	Participant monetary incentives	6 (11%)	Unclear
**D5**	Recruitment Information	Cultural considerations, incl. minority or underrepresented groups	4 (7%)	High
**A4**	Trial design	Timing of consent/ Deferred consent/ consent	3 (5%)	High
**B1**	Pre-trial planning	Trial acceptability to patients incl. patient preference	3 (5%)	High
**B11**	Pre-trial planning	Format of data collection (formally E3)	3 (5%)	High
**C9**	Trial Conduct	Cultural considerations, incl. minority or underrepresented groups	3 (5%)	Unclear
**F3**	Incentives	Participant non-monetary incentives (e.g. taxis, crèche, additional health tests)	2 (4%)	High
**G1**	Other	Decision aids	2 (4%)	High
**A3**	Trial design	Opt in/Opt out strategies	1 (2%)	High
**A7**	Trial design	Other trial designs, e.g. network analysis, indirect comparison, MAMS	1 (2%)	Unclear
**B10**	Pre-trial planning	Barriers/facilitators to recruitment (pre-study)	1 (2%)	Unclear
**C10**	Trial Conduct	Electronic Health Records	1 (2%)	Unclear
**C4**	Trial Conduct	Trial setting	1 (2%)	Unclear
**D1**	Recruitment Information	Researcher training needs	1 (2%)	High
**D7**	Recruitment Information	Trial Marketing	1 (2%)	High
**D8**	Recruitment Information	Reporting – trial wide and to Individuals	1 (2%)	Unclear
**E1**	Recruiter differences	Engagement of recruiters	1 (2%)	High
**E4**	Recruiter differences	Contact/ engagement between recruiters and patients	1 (2%)	High
**G1**	Other	Reminders	1 (2%)	High
**G1**	Other	Advance letters	1 (2%)	Unclear

a Eligible studies could report more than one domain.

**Table 4.** Frequency of retention domains reported in randomised studies of retention interventions that did not also assess recruitment.

Retention domain^a^	Domain category	Domain	Frequency N = 18	Likely impact on recruitment
B1	Participants	Reminders	8 (44%)	Low
B2	Participants	Monetary Incentives	6 (33%)	High
A3	Data collection	Data collection location and methods	4 (22%)	High
B10	Participants	Behavioural Interventions	3 (17%)	Unclear
B3	Participants	Non-monetary incentives	3 (17%)	High
A1	Data collection	Questionnaire design	1 (6%)	Low
B4	Participants	Maintaining participant engagement	1 (6%)	Low
E6	Study design	Withdrawal definition and process	1 (6%)	High

a Eligible studies could report more than one domain.

## Discussion

Updates of both the recruitment and retention databases have identified new papers which are now available on www.orrca.org.uk. Of the two research priorities, recruitment continues to attract substantially more research, but the retention literature may be more robust, with a higher percentage of randomised evaluations of retention strategies. While the number of randomised evaluations in both research priorities has been increasing over recent years,^20,22^ they continue to be a small subset of the literature and work is needed to enlarge this evidence base. Given the interconnected nature of the problem and potential solutions, dual measurement of recruitment and retention outcomes should be more frequently undertaken to increase the evidence base but more importantly to ensure strategies are of benefit to both recruitment and retention.

Although the volume of retention literature continues to grow, the focus remains heavily on recruitment with over four times the number of papers added to the recruitment database in comparison to the retention database. Newly identified literature continues to focus on types of studies most susceptible to poor recruitment and retention. Recruitment research was more likely to be undertaken in clinical studies involving drug interventions and retention research was more likely to be undertaken in mental health studies and those using behavioural interventions. While the most frequently reported recruitment and retention domains in the updates remains similar to those reported in the initial reviews,^17,18^ there are slight changes in the methods, outcomes and setting for these studies. A slightly smaller percentage of recruitment studies were conducted in North America (371/806, 46% compared to 1473/2804, 53%), with rises across all the other continents. An increased number of studies used qualitative interviews (155/806, 19% compared to 259/2804, 9%) and focus groups (63/806, 8% compared to 108/2804, 4%) which is likely connected to the growing literature exploring reasons for participation or refusal (214/806, 27% up from 566/2804, 20%). These changes are also reflected in the retention literature review. The only exception to this trend is despite seeing an increase in the use of qualitative interviews and focus groups, the number of studies assessing reasons for withdrawal has decreased (120/1167, 10% down to 10/175,6%). However, perhaps the most notable difference in the retention literature is the decreased focus on questionnaire response rate (21/175, 12% from 233/1167, 20%). This may be due to the extensive work conducted in this area historically,^25,26^ and the need to give equal attention to other attrition causes. Evaluation of retention cost has also decreased, despite this being an important consideration alongside effectiveness.^20^

Overall crossover between eligible articles for the two updates was surprisingly small, although studies returned by the literature searches were only cross-checked for inclusion in the reciprocal database if they had been categorised as randomised evaluations. Only 10 studies evaluating methodological interventions assessed both recruitment and retention outcomes. Although several randomised studies evaluated recruitment interventions that have a hypothetical impact on retention too (e.g. recruitment information, opt in/opt approach and monetary incentives), few assessed this. While many of the domains in the retention framework are unlikely to impact recruitment (e.g. monitoring approaches and site staff engagement), retention interventions frequently assessed in randomised evaluations (e.g. monetary and non-monetary incentives and data collection methods) are likely to also impact participant recruitment.

While randomised methodological research is increasing thanks to initiatives such as the PROMoting the USE of SWATs (PROMETHEUS) programme^16^ and encouragement from funding bodies,^27,28^ ongoing replication of studies is needed for both meta-analysis and to account for potential differences across health areas, populations and cultures. Current efforts to develop evidence for effective practices have been described as scatter gun.^20^ While the Cochrane reviews for recruitment and retention interventions both suggest priority assessments^20,22^ to increase the certainty of evidence, a complimentary approach would be to increase the number of evaluations. Given the reported challenges of nesting methodological research into clinical research,^16,29^ studies could maximise impact for little additional work by evaluating both recruitment and retention outcomes where possible. For example, the recent Cochrane review of retention interventions assessed five interventions exploring the effect of optimised recruitment information or a pen given at recruitment. Certainty of evidence was low and often based on a single trial (Gillies, Kearney and Keenan, 2021). If the 48 studies evaluating interventions within D2, D3 or D4 (mode and content of participant information) in this update had also assessed retention outcomes the evidence base could have been significantly increased.

While increasing the number of evaluations is an ongoing priority, dual assessment also has a more critical role considering the interconnected nature of recruitment and retention. When developing evidence for effective practices researchers need to demonstrate any impact on the reciprocal challenge to avoid recruitment being improved to the detriment of retention or vice versa. It is noted that the practicalities of embedding and reporting methodological research may mean investigators focus on either recruitment or retention. For example, the additional time needed to assess and report retention outcomes for recruitment interventions may be a limiting factor. Similarly, for retention interventions to have an impact on recruitment, they must be pre-planned and potential participants aware of the intervention before consenting to the clinical study. In practice, randomised evaluations of retention interventions may often be embedded later in clinical studies in response to suboptimal participant retention and/or data return. However, taking into account these limitations, investigators are encouraged to consider the issues raised and where possible to move away from a silo approach to methodological research.

## Conclusion

The volume of methodological literature addressing recruitment and retention challenges continues to grow as shown in the recent updates of ORRCA and ORRCA2, but randomised evaluations of recruitment/retention interventions remain a small proportion of the literature. While many nested randomised methodological studies evaluate interventions likely to impact both participant recruitment and retention, few assess both outcomes in the same study which is a methodological shortcoming given the interconnected nature of the two challenges. Where possible dual assessment of recruitment and retention outcomes should be the new standard for randomised evaluations.

## Supplemental Material

sj-docx-1-ctj-10.1177_17407745241238444 – Supplemental material for The overlap between randomised evaluations of recruitment and retention interventions: An updated review of recruitment (Online Resource for Recruitment in Clinical triAls) and retention (Online Resource for Retention in Clinical triAls) literature Supplemental material, sj-docx-1-ctj-10.1177_17407745241238444 for The overlap between randomised evaluations of recruitment and retention interventions: An updated review of recruitment (Online Resource for Recruitment in Clinical triAls) and retention (Online Resource for Retention in Clinical triAls) literature by Anna Kearney, Laura Butlin, Taylor Coffey, Thomas Conway, Sarah Cotterill, Alison Evans, Jackie Fox, Andrew Hunter, Sarah Inglis, Louise Murphy, Nurulamin M Noor, Terrie Walker-Smith and Carrol Gamble in Clinical Trials

sj-pdf-2-ctj-10.1177_17407745241238444 – Supplemental material for The overlap between randomised evaluations of recruitment and retention interventions: An updated review of recruitment (Online Resource for Recruitment in Clinical triAls) and retention (Online Resource for Retention in Clinical triAls) literature Supplemental material, sj-pdf-2-ctj-10.1177_17407745241238444 for The overlap between randomised evaluations of recruitment and retention interventions: An updated review of recruitment (Online Resource for Recruitment in Clinical triAls) and retention (Online Resource for Retention in Clinical triAls) literature by Anna Kearney, Laura Butlin, Taylor Coffey, Thomas Conway, Sarah Cotterill, Alison Evans, Jackie Fox, Andrew Hunter, Sarah Inglis, Louise Murphy, Nurulamin M Noor, Terrie Walker-Smith and Carrol Gamble in Clinical Trials

sj-pdf-3-ctj-10.1177_17407745241238444 – Supplemental material for The overlap between randomised evaluations of recruitment and retention interventions: An updated review of recruitment (Online Resource for Recruitment in Clinical triAls) and retention (Online Resource for Retention in Clinical triAls) literature Supplemental material, sj-pdf-3-ctj-10.1177_17407745241238444 for The overlap between randomised evaluations of recruitment and retention interventions: An updated review of recruitment (Online Resource for Recruitment in Clinical triAls) and retention (Online Resource for Retention in Clinical triAls) literature by Anna Kearney, Laura Butlin, Taylor Coffey, Thomas Conway, Sarah Cotterill, Alison Evans, Jackie Fox, Andrew Hunter, Sarah Inglis, Louise Murphy, Nurulamin M Noor, Terrie Walker-Smith and Carrol Gamble in Clinical Trials

sj-pdf-4-ctj-10.1177_17407745241238444 – Supplemental material for The overlap between randomised evaluations of recruitment and retention interventions: An updated review of recruitment (Online Resource for Recruitment in Clinical triAls) and retention (Online Resource for Retention in Clinical triAls) literature Supplemental material, sj-pdf-4-ctj-10.1177_17407745241238444 for The overlap between randomised evaluations of recruitment and retention interventions: An updated review of recruitment (Online Resource for Recruitment in Clinical triAls) and retention (Online Resource for Retention in Clinical triAls) literature by Anna Kearney, Laura Butlin, Taylor Coffey, Thomas Conway, Sarah Cotterill, Alison Evans, Jackie Fox, Andrew Hunter, Sarah Inglis, Louise Murphy, Nurulamin M Noor, Terrie Walker-Smith and Carrol Gamble in Clinical Trials
