# Supplementary material for: The overlap between randomised evaluations of recruitment and retention interventions: An updated review of recruitment (Online Resource for Recruitment in Clinical triAls) and retention (Online Resource for Retention in Clinical triAls) literature
Source: Clin Trials. 2024 Apr 4;21(5):640–9. doi: 10.1177/17407745241238444 (PMC11528860; doi:10.1177/17407745241238444)
Supplement: sj-docx-1-ctj-10.1177_17407745241238444 – Supplemental material for The overlap between randomised evaluations of recruitment and retention interventions: An updated review of recruitment (Online Resource for Recruitment in Clinical triAls) and retention (Online Resource for Retention in Clinical tr [file sj-docx-1-ctj-10.1177_17407745241238444.docx]

# Supplementary material: Search strategies and hand searches

1. **Revised recruitment search strategy.**

**MEDLINE(OVID)**

| 1 | (participat* or enrol* or entry* or or accru* or recruit*) ti |
| --- | --- |
| 2 | (trial* or study).ti |
| 3 | 1 AND 2 |
| 4 | Exp Patient selection/ |
| 5 | ((Participat* or recruit* or enrol*) adj4 trial*).ab,ti |
| 6 | ((Participant* or subject* or patient* or volunteer*) adj4 (recruit* or accru* or enrol*).ab,ti |
| 7 | (research).ab,ti |
| 8 | 6 AND 7 |
| 9 | Recruit*.kw |
| 10 | Recruit* adj2 (success* or difficult* or problem*).ab,ti |
| 11 | (Recruit* or enrol*) adj2 (strateg* or intervention*).ab,ti |
| 12 | ((Increas* or maximi* or promot* or improv*) adj2 (recruit* or enrol* or accru*)).ab,ti |
| 13 | ((Participant or patient or subject) adj2 (information or leaflet or video or booklet or website)) .ab,ti |
| 14 | Consent.ab,ti |
| 15 | 13 AND 14 |
| 16 | (informed consent OR Consent Process OR Consent Procedure).ab,ti,kw |
| 17 | (research OR trial*)).ab,ti,kw |
| 18 | 16 AND 17 |
| 19 | Informed consent/ |
| 20 | Consent adj5 recruit*.ab,ti |
| 21 | 3 or 4 OR 5 OR 8 Or 9 Or 10 or 11 or 12 or 15 or 18 or 19 or 20 |
| 22 | Randomized controlled trial.pt |
| 23 | Controlled clinical trial.pt |
| 24 | Randomi*ed.ab,ti,kw |
| 25 | Placebo. ab,ti,kw |
| 26 | Clinical trials as topic.sh |
| 27 | Randomly. ab,ti,kw |
| 28 | Trial*. ab,ti,kw |
| 29 | Or 22-28 |
| 30 | 21 AND 29 |
| 31 | (Trial adj2 accrual).ab,ti |
| 32 | Accrual goal*.ab,ti |
| 33 | ((Recruitment or accrual or enrol*ment) adj2 (rate* or forecast* or projection*)).ab,ti |
| 34 | 31 or 32 or 33 |
| 35 | (Research or study or trial).ab,ti |
| 36 | 34 AND 35 |
| 37 | 36 OR 30 |
| 38 | Exp animals /not humans.sh |
| 39 | 37 NOT 38 |
| 40 | Limit 39 to comment, editorial, letter or news |
| 41 | 30 NOT 40 |
| 42 | Limit to English language and year= 2018 or 2019 |

**Scopus**

| 1 | TITLE (participat* or enrol* or entry* or accru* or recruit*) |
| --- | --- |
| 2 | TITLE (trial* or study) |
| 3 | #1 AND #2 |
| 4 | TITLE-ABS-KEY ((Participat* or recruit* or enrol*) w/4 trial*) |
| 5 | (TITLE-ABS-KEY ((Participant* or subject* or patient* or volunteer*) w/4 (recruit* or accru* or enrol*))) AND  ( TITLE-ABS-KEY ( research ) ) |
| 6 | KEY (Recruit*) |
| 7 | TITLE-ABS-KEY (Recruit* w/2 (success* or difficult* or problem*)) |
| 8 | TITLE-ABS-KEY ((Recruit* or enrol*) w/2 (strateg* or intervention*)) |
| 9 | TITLE-ABS-KEY ((Increas* or maximi* or promot* or improv*) w/2 (recruit* or enrol* or accru*)) |
| 10 | TITLE-ABS-KEY ((Participant or patient or subject) w/2 (information or leaflet or video or booklet or website)) AND (TITLE-ABS-KEY(Consent)) |
| 11 | (TITLE-ABS-KEY (“Informed Consent” OR “Consent process” or “Consent procedure”)) AND (TITLE-ABS-KEY (research or trial*)) |
| 12 | TITLE-ABS-KEY (Consent w/5 recruit*) |
| 13 | #3 OR #4 OR #5 OR #6 OR #7 OR #8 OR #9 OR #10 OR #11 OR #12 |
| 14 | TITLE-ABS-KEY((clinic* w/1 trial*) OR (randomi* w/1 control*) OR (randomi* w/2 trial*) OR (random* w/1 assign*) OR (random* w/1 allocat*) OR (control* w/1 clinic*) OR (control* w/1 trial) OR placebo* OR (Quantitat* w/1 Stud*) OR (control* w/1 stud*) OR (randomi* w/1 stud*) OR (singl* w/1 blind*) or (singl* w/1 mask*) OR (doubl* w/1 blind*) OR (doubl* w/1 mask*) OR (tripl* w/1 blind*) OR (tripl* w/1 mask*) OR (trebl* w/1 blind*) OR (trebl* w/1 mask*)) |
| 15 | #13 AND #14 |
| 16 | TITLE-ABS-KEY (Trial w/2 accrual) |
| 17 | TITLE-ABS-KEY (“Accrual goal*”) |
| 18 | TITLE-ABS-KEY ((Recruitment or accrual or enrol*ment) w/2 (rate* or forecast* or projection*)) |
| 19 | #16 OR #17 OR #18 |
| 20 | TITLE-ABS-KEY (Research or study or trial) |
| 21 | #19 AND #20 |
| 22 | #15 OR #21 |
| 23 | INDEXTERMS (animals OR nonhuman) |
| 24 | DOCTYPE (cp OR ed OR le OR no OR pr) |
| 25 | #22 AND NOT (#23 OR #24) |
| 26 | LANGUAGE(English) |
| 27 | PUBYEAR IS 2018 OR 2019. |
| 28 | #25 AND #26 AND #27 |

## **Web of Science Core collection (SCI expanded and SSCI)**

| 1 | TI= (participat* or enrol* or entry* or accru* or recruit*) |
| --- | --- |
| 2 | TI= (trial* or study) |
| 3 | #1 AND #2 |
| 4 | TS=((Participat* or recruit* or enrol*) near/4 trial*) |
| 5 | TS=((Participant* or subject* or patient* or volunteer*) near/4 (recruit* or accru* or enrol*)) |
| 6 | TS= (research) |
| 7 | #5 AND #6 |
| 8 | TS= (Recruit*) |
| 9 | TS=(Recruit* near/2 (success* or difficult* or problem*)) |
| 10 | TS=((Recruit* or enrol*) near/2 (strateg* or intervention*)) |
| 11 | TS=((Increas* or maximi* or promot* or improv*) near/2 (recruit* or enrol* or accru*)) |
| 12 | TS=((Participant or patient or subject) near/2 (information or leaflet or video or booklet or website)) |
| 13 | TS= (Consent) |
| 14 | #12 AND #13 |
| 15 | TS=(“Informed Consent” or “Consent Process” or “Consent Procedure”) |
| 16 | TS=( research or trial*) |
| 17 | 15 AND 16 |
| 18 | TS=(Consent near/5 recruit*) |
| 19 | Or#3 or #4 or #7 or # 8 or #9 or #10 or #11 or #14 or #17 or #18 |
| 20 | TS=((clinic* near/1 trial*) OR (randomi* near/1 control*) OR (randomi* near/2 trial*) OR (random* near/1 assign*) OR (random* near/1 allocat*) OR (control* near/1 clinic*) OR (control* near/1 trial) OR placebo* OR (Quantitat* near/1 Stud*) OR (control* near/1 stud*) OR (randomi* near/1 stud*) OR (singl* near/1 blind*) or (singl* near/1 mask*) OR (doubl* near/1 blind*) OR (doubl* near/1 mask*) OR (tripl* near/1 blind*) OR (tripl* near/1 mask*) OR (trebl* near/1 blind*) OR (trebl* near/1 mask*)) |
| 21 | #19 AND #20 |
| 22 | TS=(Trial near/2 accrual) |
| 23 | TS=(“Accrual goal*”) |
| 24 | TS=((Recruitment or accrual or enrol$ment) near/2 (rate* or forecast* or projection*)) |
| 25 | #22 or #23 or #24 |
| 26 | TS=(Research or study or trial) |
| 27 | #25 AND #26 |
| 28 | #27 OR #21 |
| 29 | Limit to Language (English), article or review |
| 30 | (Publication year 2018 or 2019) |

**PyscINFO (EBSCO)**

| 1 | TI (participat* or enrol* or entry* or accru* or recruit*) |
| --- | --- |
| 2 | TI= (trial* or study) |
| 3 | S1 AND S2 |
| 4 | DE (“experimental recruitment”) |
| 5 | TX((Participat* or recruit* or enrol*) n4 trial*)) |
| 6 | TX((Participant* or subject* or patient* or volunteer*) n4 (recruit* or accru* or enrol*)) |
| 7 | TX (research) |
| 8 | S6 AND S7 |
| 9 | KW (Recruit*) |
| 10 | TX((Recruit* n2 (success* or difficult* or problem*)) |
| 11 | TX((Recruit* or enrol*) n2 (strateg* or intervention*)) |
| 12 | TX((Increas* or maximi* or promot* or improv*) n2 (recruit* or enrol* or accru*)) |
| 13 | TX((Participant or patient or subject) n2 (information or leaflet or video or booklet or website)) |
| 14 | TX(Consent) |
| 15 | S13 AND S14 |
| 16 | TX(“Informed Consent” or “Consent process” or “Consent procedure”) |
| 17 | TX (research or trial*) |
| 18 | S16 AND S17 |
| 19 | DE(“Informed Consent”) |
| 20 | TX(Consent n5 recruit*) |
| 21 | S3 or S4 OR S5 OR S8 Or S9 Or S10 or S11 or S12 or S15 or S18 or S19 or S20 |
| 22 | TX Double-blind |
| 23 | TX “random* assigned” |
| 24 | TX Control |
| 25 | S22 or S23 or S24 |
| 26 | S21 AND S25 |
| 27 | TX(Trial n2 accrual) |
| 28 | TX(“Accrual goal*”) |
| 29 | TX((Recruitment or accrual or enrol$ment) n2 (rate* or forecast* or projection*)) |
| 30 | S27 OR S28 OR S29 |
| 31 | TX(Research or study or trial) |
| 32 | S30 AND S31 |
| 33 | S26 OR S32 |
| 34 | Limit to animal |
| 35 | S33NOT S34 |
| 36 | Limit to Language (English) and (Publication year 2018 or 2019) |

**CINHAL Plus ( EBSCO)**

| 1 | TI (participat* or enrol* or entry* or accru* or recruit*) |
| --- | --- |
| 2 | TI (trial* or study) |
| 3 | #1 AND #2 |
| 4 | TX((Participat* or recruit* or enrol*) n4 trial*)) |
| 5 | TX((Participant* or subject* or patient* or volunteer*) n4 (recruit* or accru* or enrol*)) |
| 6 | TX (research) |
| 7 | S5 AND S6 |
| 8 | TX (Recruit*) |
| 9 | TX((Recruit* n2 (success* or difficult* or problem*)) |
| 10 | TX((Recruit* or enrol*) n2 (strateg* or intervention*)) |
| 11 | TX((Increas* or maximi* or promot* or improv*) n2 (recruit* or enrol or accru*)) |
| 12 | TX((Participant or patient or subject) n2 (information or leaflet or video or booklet or website)) |
| 13 | TX(Consent) |
| 14 | S12 AND S13 |
| 15 | TX(“Informed Consent” or “Consent process” or “Consent procedure”) |
| 16 | TX (research or trial*) |
| 17 | S15 AND S16 |
| 18 | TX(Consent n5 recruit*) |
| 19 | S3 or S4 OR OR S7 OR S8 OR S9 Or S10 or S11 or S14 or S17 or S18 |
| 20 | PT Clinical trial |
| 21 | MH “treatment outcomes” |
| 22 | TX randomi#ed |
| 23 | #20 or #21 or #22 |
| 24 | #19 and #23 |
| 25 | TX(Trial n2 accrual) |
| 26 | TX(“Accrual goal*”) |
| 27 | TX((Recruitment or accrual or enrol$ment) n2 (rate* or forecast* or projection*)) |
| 28 | #25 OR #26 OR #27 |
| 29 | TX(Research or study or trial) |
| 30 | #28 and #29 |
| 31 | #24 or #30 |
| 32 | Limit to Language (English), (Publication year 2018 or 2019) |

**Cochrane Library**

| 1 | (participat* or enrol* or entry* or accru* or recruit*):ti |
| --- | --- |
| 2 | (trial* or study):ti |
| 3 | #1 AND #2 |
| 4 | Exp Patient selection/ |
| 5 | ((Participat* or recruit* or enrol*) near/4 trial*):ab,ti |
| 6 | (Participant* or subject* or patient* or volunteer*) near/4 (recruit* or accru* or enrol*):ab,ti |
| 7 | (research):ab,ti |
| 8 | #6 AND #7 |
| 9 | Recruit*:kw |
| 10 | Recruit* near/2 (success* or difficult* or problem*):ab,ti |
| 11 | (Recruit* or enrol*) near/2 (strateg* or intervention*):ab,ti |
| 12 | (Increas* or maximi* or promot* or improv*) near/2 (recruit* or enrol or accru*):ab,ti |
| 13 | (Participant or patient or subject) near/2 (information or leaflet or video or booklet or website):ab,ti |
| 14 | Consent:ab,ti |
| 15 | #13 AND #14 |
| 16 | ((informed consent OR Consent Process OR Consent Procedure):ab,ti,kw |
| 17 | (research OR trial*):ab,ti,kw |
| 18 | #16 AND #17 |
| 19 | Informed consent/ |
| 20 | Consent near/5 recruit*:ab,ti |
| 21 | #3 or #4 or #5 or #8 or #9 or #10 or #11 or #12 or #15 or #18 or #19 or #20 |
| 22 | Randomized controlled trial:pt |
| 23 | Controlled clinical trial:pt |
| 24 | Randomi*ed:ab,ti,kw |
| 25 | Placebo:ab,ti,kw |
| 26 | Randomly;ab,ti,kw |
| 27 | Trial*:ab,ti,kw |
| 28 | #22 or #23 or #24 or #25 or #26 or #27 |
| 29 | #21 AND #28 |
| 30 | (Trial near/2 accrual):ab,ti |
| 31 | (“Accrual goal*”):ab,ti |
| 32 | (Recruitment or accrual or enrol*ment) near/2 (rate* or forecast* or projection*):ab,ti |
| 33 | OR30-32 |
| 34 | (Research or study or trial):ab,ti |
| 35 | #33 AND #34 |
| 36 | #29 or #35 |
| 37 | Limited to trials, reviews and publication year 2018 or 2019 |

1. **Hand searches for recruitment database**
2. Treweek S, Pitkethly M, Cook J, Fraser C, Mitchell E, Sullivan F, et al. Strategies to improve recruitment to randomised trials. Cochrane Database Syst Rev. 2018;2(2):MR000013.
3. Crocker JC, Ricci-Cabello I, Parker A, Hirst JA, Chant A, Petit-Zeman S, et al. Impact of patient and public involvement on enrolment and retention in clinical trials: systematic review and meta-analysis. BMJ-British Medical Journal. 2018;363.
4. Delaney H, Devane D, Hunter A, Hennessy M, Parker A, Murphy L, et al. Limited evidence exists on the effectiveness of education and training interventions on trial recruitment; a systematic review. J Clin Epidemiol. 2019;113:75-82.
5. **Hand searches for retention database**
6. Crocker JC, Ricci-Cabello I, Parker A, Hirst JA, Chant A, Petit-Zeman S, et al. Impact of patient and public involvement on enrolment and retention in clinical trials: systematic review and meta-analysis. BMJ-British Medical Journal. 2018;363.
7. Liu Y, Pencheon E, Hunter RM, Moncrieff J, Freemantle N. Recruitment and retention strategies in mental health trials - A systematic review. Plos One. 2018;13(8):e0203127-e.
8. Blumenberg C, Barros AJD. Response rate differences between web and alternative data collection methods for public health research: a systematic review of the literature. International Journal of Public Health. 2018;63(6):765-73.
9. van Gelder MMHJ, Vlenterie R, IntHout J, Engelen LJLPG, Vrieling A, van de Belt TH. Most response-inducing strategies do not increase participation in observational studies: a systematic review and meta-analysis. Journal of clinical epidemiology. 2018;99:1-13.
