# Supplementary figures and images for: The overlap between randomised evaluations of recruitment and retention interventions: An updated review of recruitment (Online Resource for Recruitment in Clinical triAls) and retention (Online Resource for Retention in Clinical triAls) literature
Source: Clin Trials. 2024 Apr 4;21(5):640–9. doi: 10.1177/17407745241238444 (PMC11528860; doi:10.1177/17407745241238444)

# Recruitment Research Domains

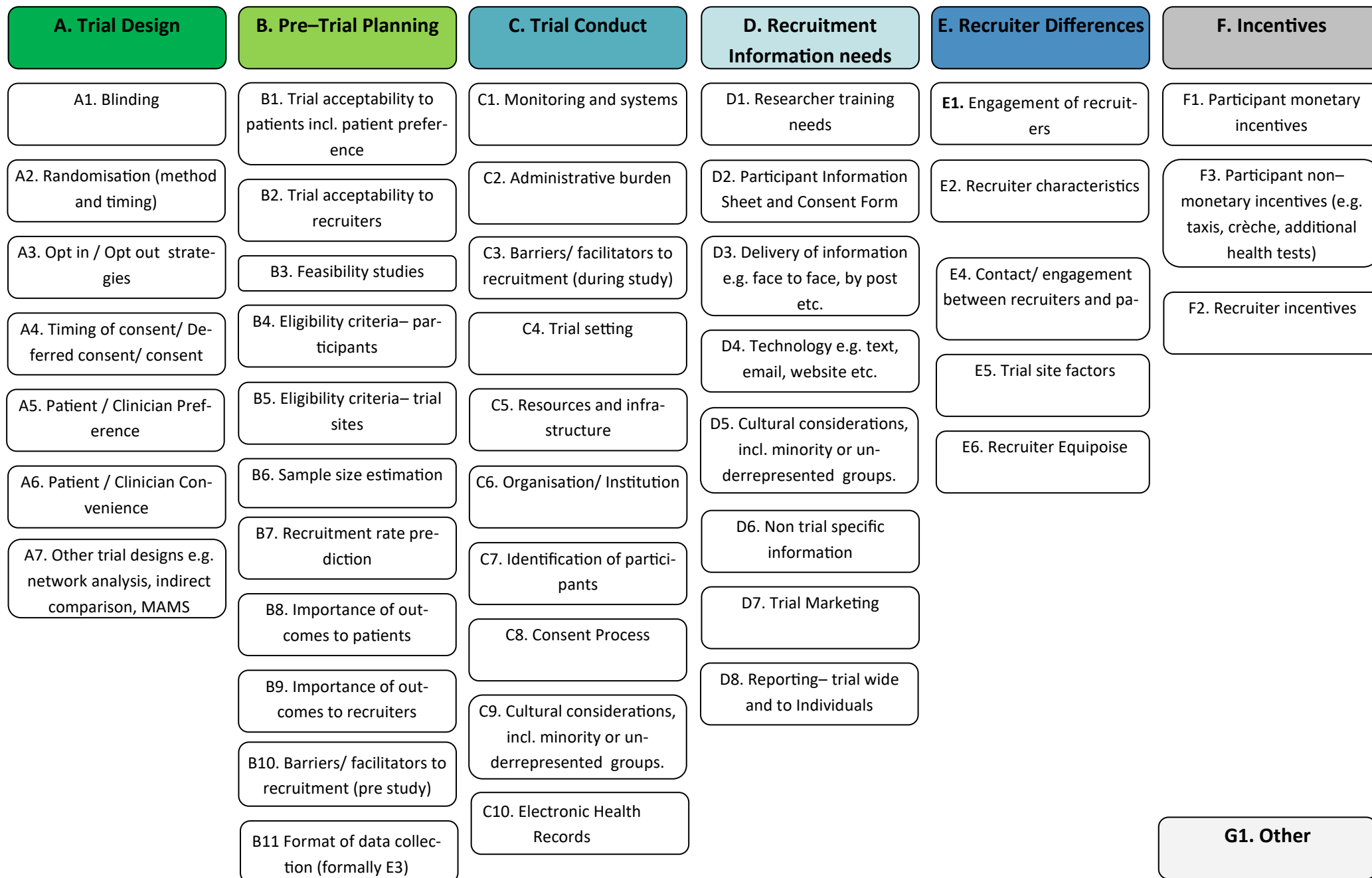

Supplement: sj-pdf-2-ctj-10.1177_17407745241238444 – Supplemental material for The overlap between randomised evaluations of recruitment and retention interventions: An updated review of recruitment (Online Resource for Recruitment in Clinical triAls) and retention (Online Resource for Retention in Clinical tri [file sj-pdf-2-ctj-10.1177_17407745241238444.pdf]
