# Supplementary material for: The overlap between randomised evaluations of recruitment and retention interventions: An updated review of recruitment (Online Resource for Recruitment in Clinical triAls) and retention (Online Resource for Retention in Clinical triAls) literature
Source: Clin Trials. 2024 Apr 4;21(5):640–9. doi: 10.1177/17407745241238444 (PMC11528860; doi:10.1177/17407745241238444)
Supplement: sj-pdf-3-ctj-10.1177_17407745241238444 – Supplemental material for The overlap between randomised evaluations of recruitment and retention interventions: An updated review of recruitment (Online Resource for Recruitment in Clinical triAls) and retention (Online Resource for Retention in Clinical tri [file sj-pdf-3-ctj-10.1177_17407745241238444.pdf]

Retention Research Domains

ORRCA
www.orrca.org.uk

A. Data Collection	B. Participants	C. Sites and Site Staff	D. Central Study Management	E. Study Design
A1. Questionnaire design	B1. Reminders	C1. Reminders	D1. Monitoring approach	E1. Choice of study outcomes
A2. Data collection frequency/ timing	B2. Monetary incentives	C2. Monetary incentives	D2. Resources and infrastructure	E2. Feasibility studies
A3. Data collection location and method	B3. Non–monetary incentives	C3. Non–monetary incentives	D3. Organisation/ institution	E3. Impact of recruitment
A4. Routine Data (ONS, HES, Electronic Record)	B4. Maintaining participant engagement	C4. Maintaining staff engagement	D4. PPI / CBPR	E4. Randomisation method
A5. Data collection during routine care	B5. Acceptability of protocol (incl. patient burden)	C5. Acceptability of protocol (incl. admin burden)	D5. CRF design	E5. Blinding and treatment preference
A6. Who collects the data	B6. Participant factors	C6. Trial site factors	D6. Study Identity / Branding	E6. Withdrawal definition and process
	B7. Supporting participation (creche, expenses)	C7. Resources and infrastructure		E7. Run in period
	B8. Contact Information	C8. Site selection		E8. Estimating attrition / sample size calculation
	B9. Cultural Considerations	C9. Training		E9. Other trial design
	B10. Behavioural Interventions	C10. Monitoring visits		E10. Trial Setting
	B11 Relationship with clinical staff			
	B12. Motivations and experience.			

G1. Other
