# Supplementary material for: The overlap between randomised evaluations of recruitment and retention interventions: An updated review of recruitment (Online Resource for Recruitment in Clinical triAls) and retention (Online Resource for Retention in Clinical triAls) literature
Source: Clin Trials. 2024 Apr 4;21(5):640–9. doi: 10.1177/17407745241238444 (PMC11528860; doi:10.1177/17407745241238444)
Supplement: sj-pdf-4-ctj-10.1177_17407745241238444 – Supplemental material for The overlap between randomised evaluations of recruitment and retention interventions: An updated review of recruitment (Online Resource for Recruitment in Clinical triAls) and retention (Online Resource for Retention in Clinical tri [file sj-pdf-4-ctj-10.1177_17407745241238444.pdf]

Supplementary material: Additional Figures and tables

Supplementary Table 1: Cohort of new papers:

Category[1]	Type	Recruitment (n=806)	Retention n=175
Host	RCT	517 (64%)	102 (58%)
	Cohort	103 (13%)	41 (23%)
	Unknown	99 (12%)	13 (7%)
	Early Phase Trials (Not Randomised)	36 (4%)	1 (1%)
	Survey	35 (4%)	8 (5%)
	Cluster RCT	24 (3%)	13 (7%)
	Biobank	23 (3%)	0 (0%)
	Other	10 (1%)	0 (0%)
	Registry	9 (1%)	3 (2%)
	Factorial RCT	8 (1%)	2 (1%)
	Case Control	7 (1%)	1 (1%)
	Interview	7 (1%)	1 (1%)
	Non-Randomised Pilot/ Feasibility Study	4 (<1%)	1 (1%)
	Patient Preference	4 (<1%)	2 (1%)
	Crossover RCT	3 (<1%)	0 (0%)
	Focus Group	3 (<1%)	0 (0%)
	Data Linkage	1 (<1%)	0 (0%)
	N of 1	1 (<1%)	0 (0%)
Location	N. America	371 (46%)	68 (39%)
	Europe	219 (27%)	55 (31%)
	Unknown	138 (17%)	30 (17%)
	Australasia	72 (9%)	14 (8%)
	Asia	42 (5%)	9 (5%)
	Africa	33 (4%)	16 (9%)
	S. America	15 (2%)	2 (1%)
Recruitment setting	Secondary or Tertiary Care	276 (34%)	56 (32%)
	Other	253 (31%)	39 (22%)
	Unknown	234 (29%)	64 (37%)
	Primary Care	88 (11%)	17 (10%)
	Emergency Care	39 (5%)	4 (2%)
	Intensive Care	14 (2%)	3 (2%)
	School	9 (1%)	6 (3%)
Health area	Cancer	212 (26%)	21 (12%)
	Unknown	103 (13%)	12 (7%)
	Mental Health	98 (12%)	34 (19%)
	Infection	80 (10%)	20 (11%)
	Neurological	79 (10%)	17 (10%)

	Cardiovascular	67 (8%)	6 (3%)
	Reproduction	57 (7%)	19 (11%)
	Generic	46 (6%)	13 (7%)
	Metabolic and Endocrine	38 (5%)	7 (4%)
	Stroke	26 (3%)	5 (3%)
	Musculoskeletal	24 (3%)	14 (8%)
	Inflammatory and Immune	23 (3%)	1 (1%)
	Injuries	23 (3%)	21 (12%)
	Respiratory	23 (3%)	10 (6%)
	Renal	10 (1%)	5 (3%)
	Congenital	9 (1%)	0 (0%)
	Oral and Gastrointestinal	7 (1%)	1 (1%)
	Skin	5 (1%)	2 (1%)
	Blood	4 (<1%)	0 (0%)
	Eye	3 (<1%)	1 (1%)
	Ear	2 (<1%)	1 (1%)
	Other	2 (<1%)	1 (1%)
Health intervention	Unknown	295 (37%)	27 (15%)
	Drug	181 (22%)	19 (11%)
	N/A	119 (15%)	41 (23%)
	Behavioural	93 (12%)	42 (24%)
	Surgery	36 (4%)	11 (6%)
	Other	34 (4%)	13 (7%)
	Care Pathway	31 (4%)	17 (10%)
	Vaccine	27 (3%)	6 (3%)
	Physical	21 (3%)	11 (6%)
	Screening	15 (2%)	3 (2%)
	Medical Device	13 (2%)	4 (2%)
	Complimentary Therapy	3 (<1%)	2 (1%)
	Diagnostic	3 (<1%)	0 (0%)
	Older Than 18	436 (54%)	107 (61%)
Age	Older Than 60	275 (34%)	52 (30%)
	Unknown	233 (29%)	41 (23%)
	Younger Than 16	136 (17%)	28 (16%)
	16-18 years	80 (10%)	19 (11%)

[1]Articles were coded against all relevant options within each category.

Supplementary Figure 1: Recruitment domains for all papers and those categorised as randomised evaluation

Notes: Full names of the domains are available in the domain frameworks which can be accessed via the supplementary material or online at www.orrca.org.uk. Articles were coded against all relevant domains. Domain E3 was recategorized into C10 and so all data is reported underE3 in the update.

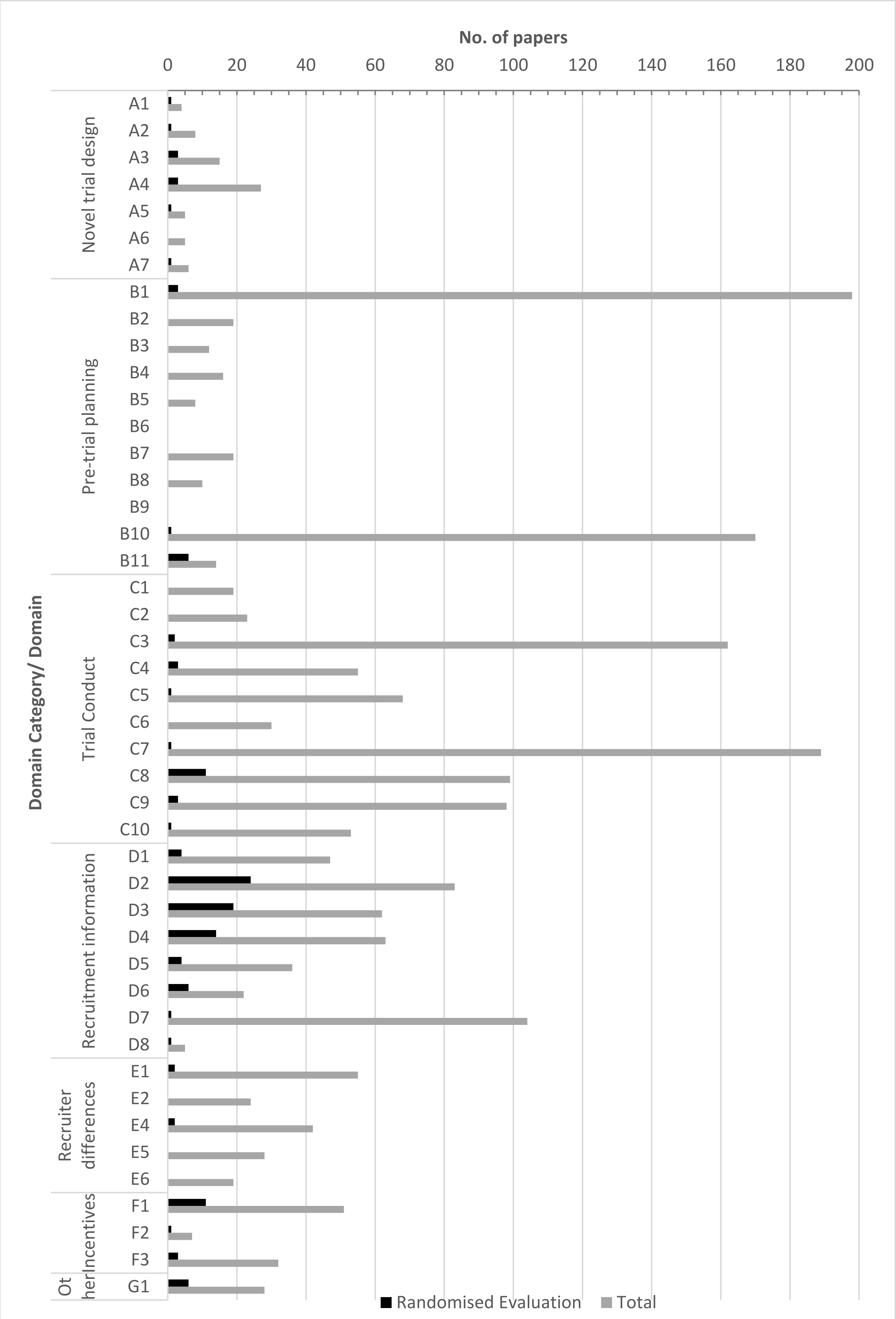

Supplementary Figure 2: Retention domains for all papers and those categorised as randomised evaluation

Notes: Full names of the domains are available in the domain frameworks which can be accessed via the supplementary material or online at www.orrca.org.uk. Articles were coded against all relevant domains

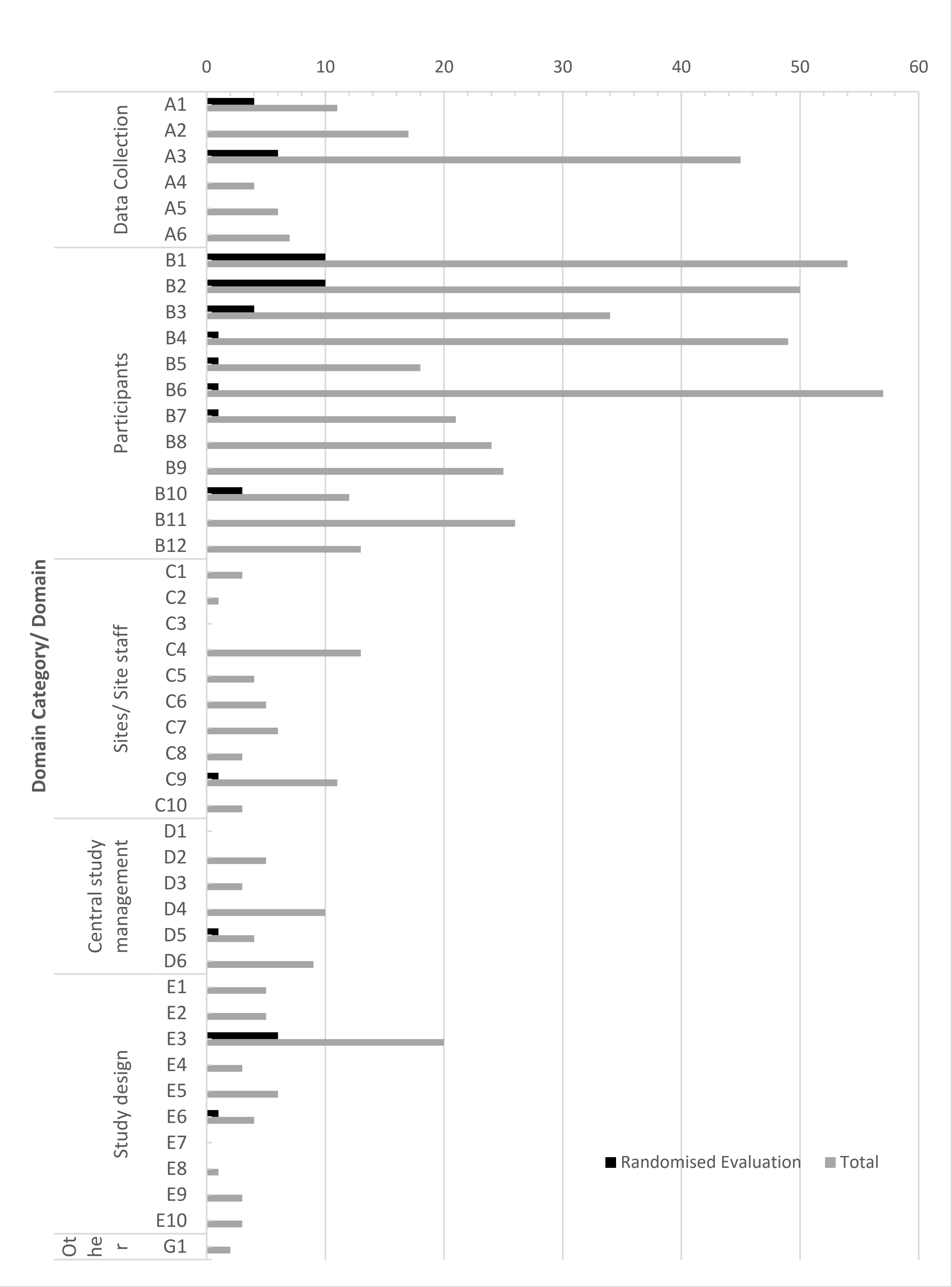

Supplementary Table 2: Systematic reviews categorised as randomised evaluations that appear in both database

ID	Focus	Outcomes		Domains	
		Recruitment	Retention	Recruitment	Retention
2469	Impact of Patient and public involvement on recruitment and retention	No. recruited	No. retained	G1	B7; D5; E3
3067	Recruitment / retention strategies for observational studies	No. recruited, Questionnaire RR	No. retained, Questionnaire RR	F1; F2; F3; B11	A1; B1; B2; B3
4228	Recruitment / retention strategies for mental health trials	No. recruited, Questionnaire RR	No. retained, Questionnaire RR, Retention Cost	C3; C4; C5; C8; D2; F1	A1; B1; B2
